# Complete Electrolytic Plastron Recovery in a Low Drag Superhydrophobic Surface

**DOI:** 10.1021/acsomega.0c03466

**Published:** 2021-01-28

**Authors:** Ben P. Lloyd, Philip N. Bartlett, Robert J. K. Wood

**Affiliations:** † National Centre for Advanced Tribology at Southampton (nCATS), University of Southampton, Southampton, SO17 1BJ, U.K.; ‡ Chemistry, University of Southampton, Southampton, SO17 1BJ, U.K.

## Abstract

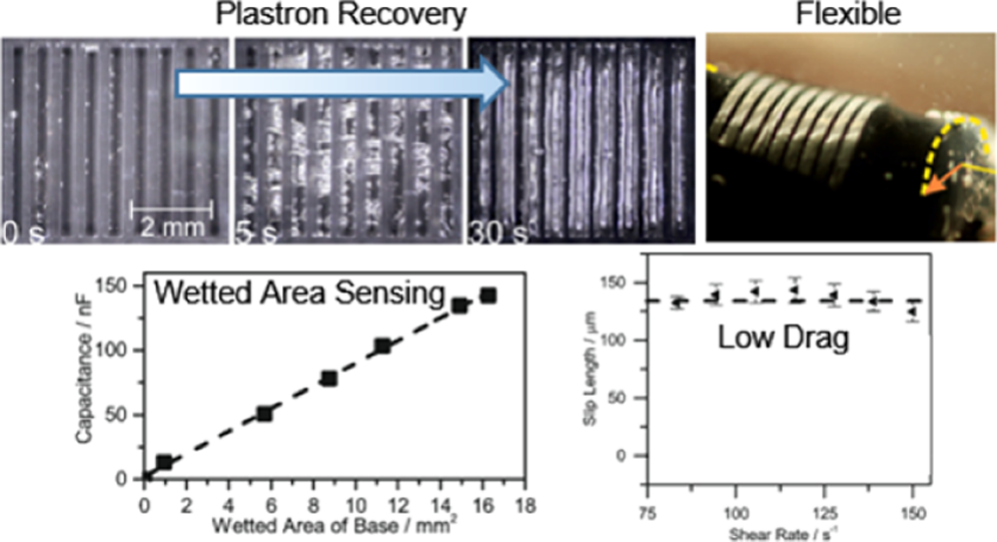

We present a superhydrophobic surface capable of recovering the lubricious gas layer known as the “plastron” from a fully wetted state underwater. It is shown that full plastron recovery is possible without a second layer of structural hierarchy, which is prone to irreversible wetting transitions. This allows us to use a cheap, fast, and potentially scalable method to fabricate the surface from silicone and carbon black in a molding process. We demonstrate plastron recovery from the fully wetted state and immediate plastron recovery after pressure-induced wetting transitions. The wetting state can be measured remotely and quickly by measuring the capacitance. The slip length is measured as ∼135 μm, agreeing well with the theory given the geometry of the surface. The ability of the surface to conform to small radii of curvature and withstand damage from loading is also demonstrated. The work presented here could allow superhydrophobic surfaces to reduce drag on ships and in pipes where the plastron would otherwise rapidly dissolve.

## Introduction

As a large ship passes through the water, up to 70% of the fuel is burned to overcome skin friction drag acting on the hull^1^ and in hydroelectric facilities skin frictional drag can cause energy losses of up to 20%.^2^ Superhydrophobic surfaces can significantly reduce skin friction drag in laminar^3^ and turbulent flows.^4^ When submerged in water, a lubricious gas layer is trapped on the surface by hydrophobic surface features, which leads to effective slip of water over the surface.^5^ Superhydrophobic surfaces may be able to minimize these losses in real-world scenarios;^6^ however, before this technology could be adopted, there are several problems which must be solved.

A significant obstacle for prolonged drag reduction using superhydrophobic surfaces is the inherent fragility of the gas layer trapped between the liquid and the solid also known as a “plastron.” It can be lost through excessive pressure difference between itself and the water, which leads to an initial compression^7^ and subsequent dissolution.^8^ Once lost, the plastron is hard to recover as generally the wetted state is thermodynamically more favorable.

**Figure 1:**
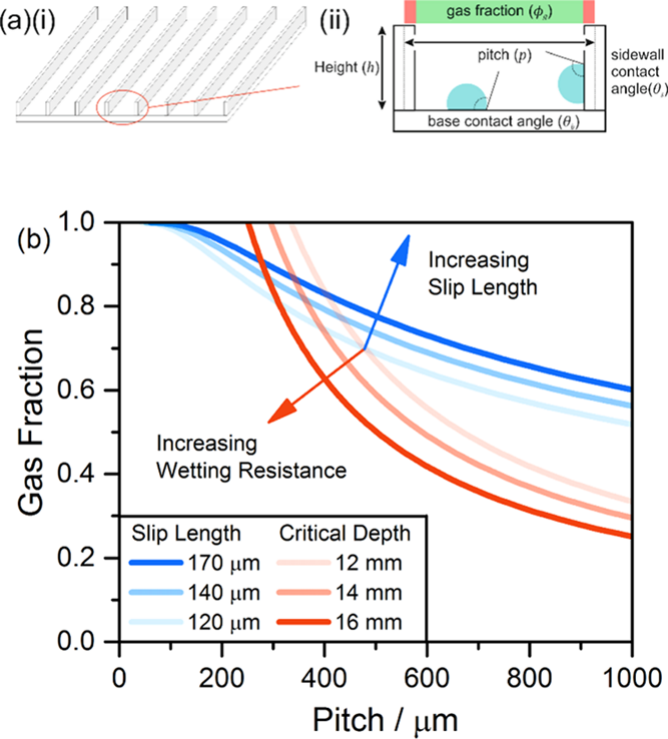
(a(i)) Schematic of a surface consisting of parallel channels. (a(ii)) Schematic of one channel with key parameters identified. (b) Graph showing the dependence of critical depth and slip length on the gas fraction and pitch of a superhydrophobic surface consisting of parallel channels. It is not possible to have both large critical depth and slip length. The isopleths of critical depth are calculated using eq 1 assuming a sidewall contact angle of θ_s_ of 110° and plotted in red. The isopleths of slip length are calculated using eq 2 and plotted in blue.

A convenient measure of resistance to wetting is the critical depth, *d*_crit_. This is the depth at which water pressure overcomes capillary pressure and dissolution of the plastron starts to occur. To make a superhydrophobic surface more resistant to wetting, the geometry should be altered to reduce the pitch, *p* (the center to center spacing between channel walls) and reduce the gas fraction, ϕ_g_ (the fraction of the surface occupied by the plastron averaged over a large area of the surface), as shown in eq 1.^9^
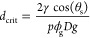
1where θ_s_ is sidewall contact angle, γ is the surface tension, *D* is the liquid density, and *g* is the acceleration due to gravity. Equation 1 is valid for the normal case where dissolved gases in the water are in equilibrium with the atmosphere. Figure 1a(i) shows a superhydrophobic surface made of parallel channels and (ii) identifies the defining parameters.

However, this surface with a small pitch and gas fraction which has good wetting resistance would be very poor at generating slip, the streamwise movement of the water at the liquid/gas interface. A large slip length, δ, is achieved with a large pitch and large gas fraction as shown in eq 2.^10^
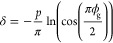
2This dichotomy is shown graphically in Figure 1, where the isopleths of slip length and critical depth are plotted as a function of gas fraction and pitch for a surface consisting of parallel channels, which has been shown to be an effective superhydrophobic surface geometry for drag reduction.

Although there is a benefit in choosing a large gas fraction and small pitch to maximize both properties, even in this ideal case, critical depth and slip length still oppose one another. There is no point on this gas fraction/pitch landscape where we can achieve a desirable outcome of both large slip length and large critical depth; it will always be a compromise.

While passive methods such a sidewall texturing, where nanosized structures are added to the sides of microsized structures, have shown that plastron loss from dissolution and pressure can be delayed (which acts to move the isopleths of critical depth upwards and rightwards in Figure 1 by increasing θ_s_ in eq 1), ultimately plastron loss cannot be avoided.^11^ Passive dewetting of conical pores is possible at ambient pressures. However, this behavior requires very small pores and thus would not generate a large slip length.^12^

If we accept that there is no escaping the dichotomy presented in Figure 1, a solution could be to employ a system where the plastron is actively maintained. If one had the ability to replenish the plastron, the surface could be designed to prioritize large slip length over wetting resistance and accept that while the plastron is easily lost it could also be recovered on demand. Methods to generate a new plastron when submerged under water include chemical decomposition of hydrogen peroxide,^13^ thermally induced supersaturation of gases in surrounding water,^14^ thermal vaporization,^15^ pumping of pressurized air,^16^ and electrolysis.^17,18^ Very recently, the electrolysis has been shown to be powered by the corrosion of magnesium avoiding the use of an external power supply, however with the downside of having finite use due to consumption of magnesium.^19^

**Figure 2:**
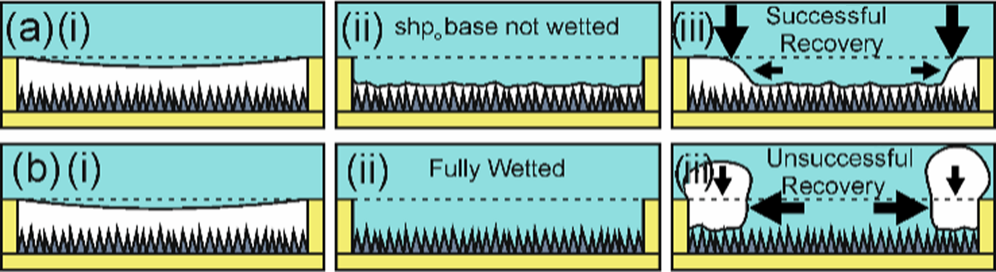
Successful (a) and unsuccessful (b) plastron recovery in a channel with a superhydrophobic base viewed looking through the side of a channel, showing that when the nanostructured base becomes fully wetted, the plastron cannot be recovered. (a(i)) shows the native gas film present in the channel. (a(ii)) the channel is wetted; however, the plastron of the nanostructured base remains intact. (a(iii)) gas is generated, and as the forces opposing lateral growth are less than forces opposing vertical growth, the plastron fills the channel. (b(i)) Starting again with the native gas film present in the channel. (b(ii)), both the channel and the nanostructured base are wetted. (b(iii)) when gas is generated, the forces opposing lateral growth are greater than forces opposing vertical growth so the plastron is lost as bubbles from the top of the channel.

To this end, Lee and Kim used electrolytic gas evolution as an effective means of plastron replenishment.^17^ To achieve successful plastron recovery, they identified that the capillary pressures acting on the evolved gas must be lower for lateral spreading along the channels than vertical growth out of them. If this condition was not met, the newly generated gas was lost as bubbles exiting the top of the surface. A caveat to their surface design was that a nanosized superhydrophobic base must be used between the microsized superhydrophobic structures and that the plastron of this nanosized superhydrophobic base must stay intact for full plastron to be recovered, Figure 2. This meant that the plastron could not be recovered when the surface was fully wetted. If for some reason this became wetted, e.g., from a prolonged period without active gas generation, large pressures generated by waves or water with low dissolved gas concentration, then the surface would not be able to recover, thus irreversibly losing its low drag properties.

Another more practical problem for large-scale drag reduction is how one might make a surface, which can be produced in large areas at reasonable cost so that it could coat something as large as a ship. To give an appreciation of the areas required, a Maersk triple E has a wetted area of 27 500 m^2^.^20^ Most low drag superhydrophobic surfaces have been made using cleanroom techniques on the order of centimeters squared, which could not be scaled to produce large areas at low cost. Furthermore, these surfaces are often made of silicon which would not conform to the hull and surface features could be easily damaged.

In this paper, we demonstrate that the need for a nanostructured base as used by Lee and Kim^17^ can be avoided in some situations. This has the significant benefit that the plastron can be recovered by electrolysis even when the surface has been fully wetted and therefore ensure that drag reduction will not be compromised, which has not previously been demonstrated. Further to this, the fact that we do not need to use a nanostructured base means that the fabrication of our surface is easy, economical, and fast to make. We also demonstrate: immediate recovery when the plastron is partially removed by an impinging water jet, fast and accurate sensing of the wetting state by measuring surface capacitance, measurement of the slip length and the surface’s mechanical flexibility, and damage resistance to loading.

## Results and Discussion

### Plastron Recovery without a Superhydrophobic Base

**Figure 3:**
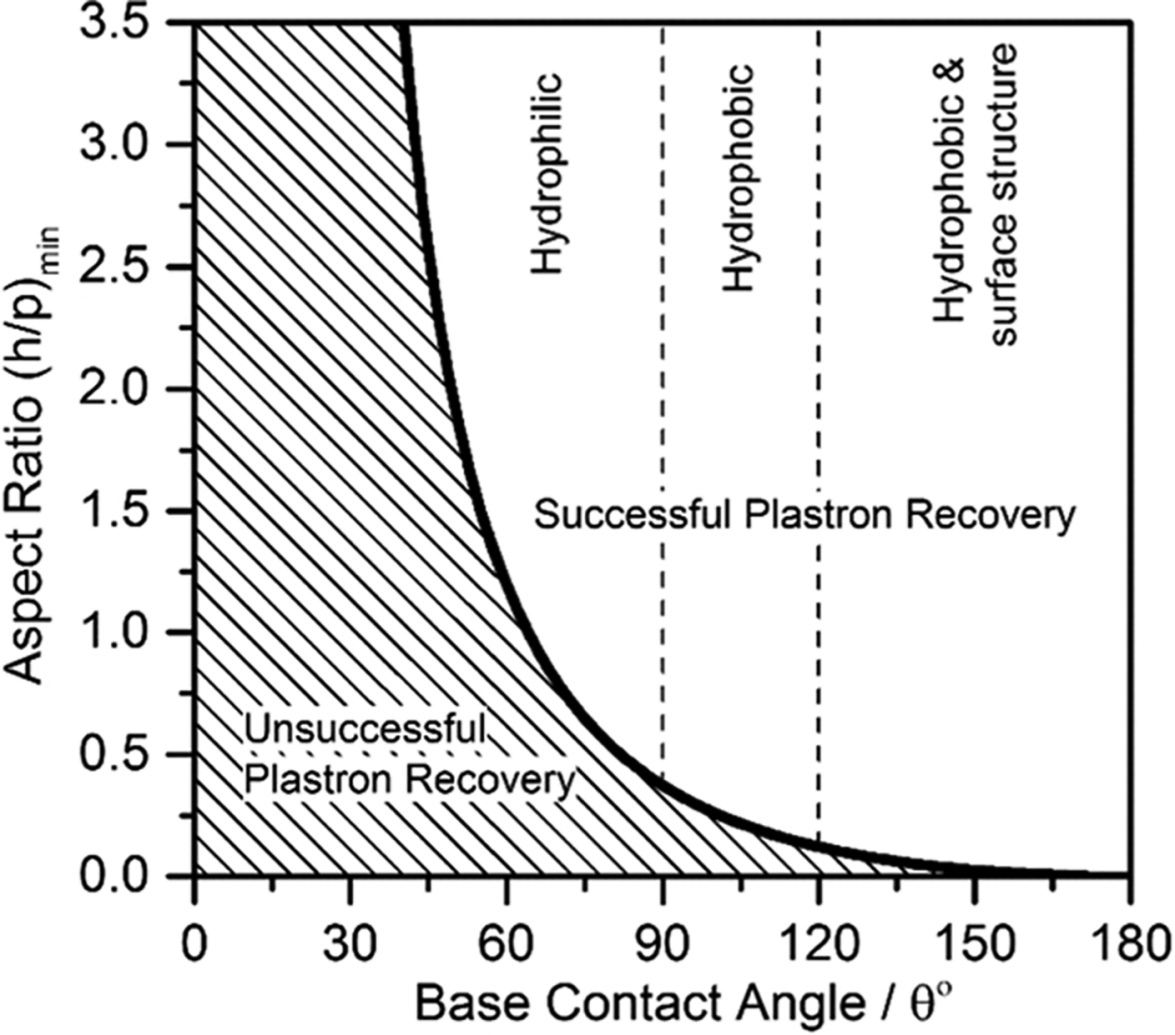
Regimes of successful and unsuccessful plastron recovery shown as the minimum channel height to pitch ratio as a function of the base contact angle. Plotted using eq 3 for a sidewall contact angle of 110°. Plastron recovery should be possible even with a hydrophilic base at reasonable channel height to pitch ratios.

To successfully recover the plastron, the capillary pressure must be lower for lateral spreading than vertical spreading, otherwise the newly generated gas will be lost as bubbles. This criterion can be expressed in terms of the minimum channel height to pitch ratio, which would lead to desired capillary pressures, as shown in eq 3.^17^ In the work of Lee and Kim, *h*/*p*_min_ was only considered as a function of gas fraction.
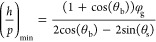
3Here, in Figure 3, we show eq 3, the minimum height/pitch ratio as a function of the base contact angle for typical values of ϕ_g_ = 0.9 and θ_s_ = 110°. This clearly shows that horizontal spreading is very much possible without the need for a superhydrophobic base and is possible even using a hydrophilic base and reasonable h/p aspect ratios.

### Fabrication of Surface

**Figure 4:**
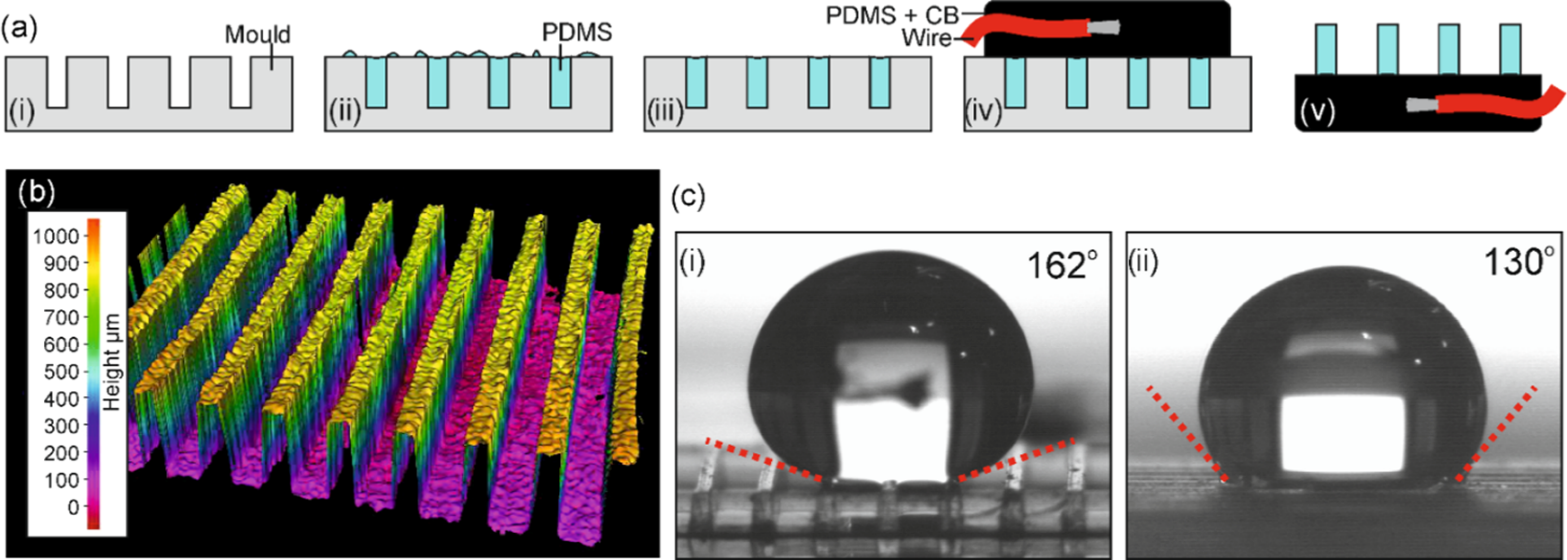
(a) Fabrication of the surface using a molding process. (i) Mold is created. (ii) Liquid PDMS fills the channels in the mold and is left to cure. (iii) Excess PDMS is cleaned from the top of the mold. (iv) PDMS and carbon black mixture with embedded wire covers the top of the mold and left to cure. (v) Surface is demolded. (b) Optical height image of the surface. (c) Contact angle measurements of water drops on the surface. (i) Measuring the contact angle perpendicular to the channel gives a contact angle of 162° and (ii) parallel to the channels gives a contact angle of 130°.

Removing the requirement for a superhydrophobic base allows us to use a molding process to generate the surface, shown schematically in Figure 4a. An acrylic (lucite) mold is used where channels have been machined using a CNC router. Poly(dimethylsiloxane) (PDMS) is used to fill the channels in the mold by pulling the uncured liquid PDMS over the mold using a rubber “squigie”; these regions become the walls of the channels. After curing, the mold is lightly polished with water and a fine polishing cloth to clean away any excess PDMS remaining on the top of the mold. A layer of conductive PDMS is applied over the top of the PDMS-filled channels and a wire is embedded; this is the base of the surface. After curing, the surface is demolded and the exposed areas of the conductive silicone are covered by PDMS to be electrically insulative. Figure 4b shows a three-dimensional (3D) microscopic image of the resulting surface.

Poly(dimethylsiloxane) (Sylgard 184 from Dow) is used for the sidewall for its high contact angle (110°) and electrically insulative properties. For the base, we used a poly(dimethylsiloxane) and carbon black composite (Vulcan XC-72R 20% by weight in Sylgard 184), which had a reasonable receding contact angle of 65° and good electronic conductivity. We used a wall thickness of 200 μm at a pitch of 600 μm with a height of 850 μm. This gives a gas fraction of 0.66. From eq 3, the *h*/*p*_min_ is 0.92, the h/p of our surface was 1.42, capable of recovery if the expanding plastron was pinned to a maximum of 55°. Using eq 1, the critical depth is 12.5 mm. Using eq 2, the slip length is 134 μm.

Interestingly, we see that the contact angle on this surface is heavily dependent on the orientation to the channels, Figure 4c. We observe a contact angle of 162° when measuring perpendicular to the channels and 130° when measuring in the parallel direction, a similar phenomenon to the crystal face-dependent spreading in earlier work.^21^

Our fabrication method overcomes two key challenges when making such a surface. First, there is good selectivity over where the two components are located; it is important that no insulative PDMS covers the conductive PDMS. During fabrication, when filling the channels in the mold, a very thin layer of insulative PDMS also covers the top of the mold. This was easily sheared off with light polishing without removing the PDMS in the channels ensuring that the conductive PDMS was fully exposed on the base. Second, there is excellent adhesion between the walls and the base—this was hard to achieve due to the necessarily low surface energy of the wall material; however, this was not a problem when using a silicone for both wall and base. Furthermore, silicones are already established as a “foul release” type antifouling coatings on ships, *e.g*. Hempel Silic One, and therefore represent a reasonable material, choice for this application.

By basing the fabrication on a molding process, the time-consuming process of microstructuring the surface is only required once. Replicas of the mold are fast to produce and do not lead to degradation of the mold. Several samples were made using the method described here all of which behaved similarly; one of these samples was characterized in depth to assess full plastron recovery, immediate partial plastron recovery, and sensing of the wetting state.

### Full Plastron Recovery

The dewetting performance of the surface was investigated by submerging in 10 mm of 0.06 M NaCl solution and displacing the plastron. A potential difference of −10 V vs Pt wire was applied to the surface to run the electrolysis reaction, and platinum gauze was used at the counter electrode. The process was filmed from above using a camera mounted to a macroscope.

**Figure 5:**
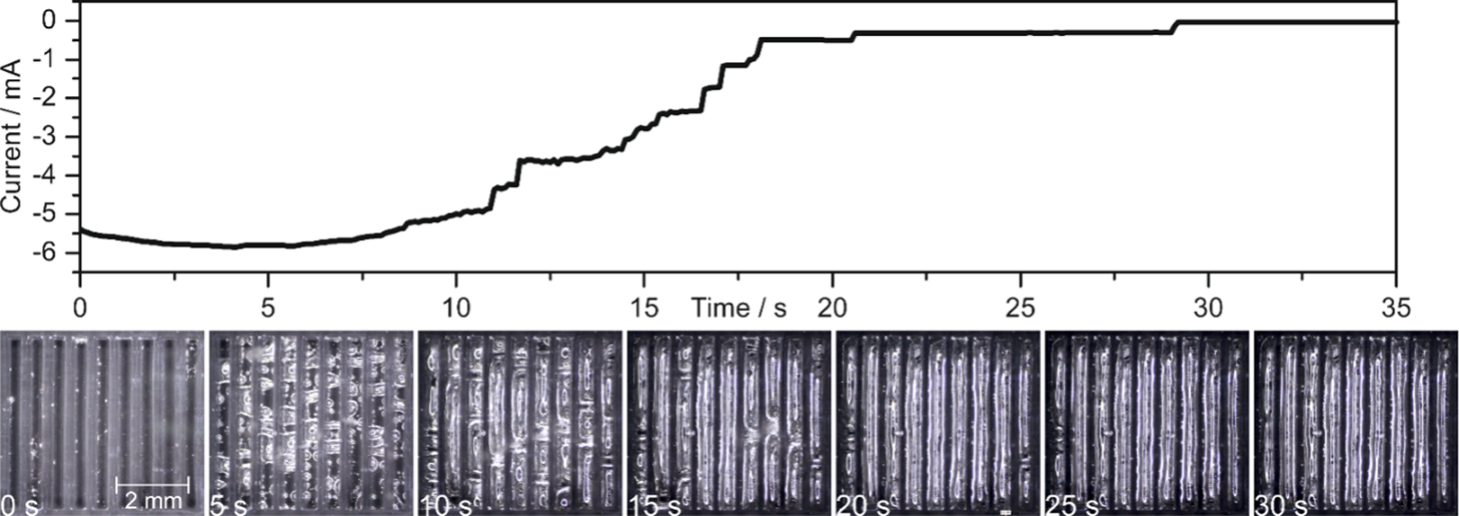
Full plastron recovery from a fully wetted state. The graph shows the current transient when a potential of −10 V is applied to the surface. Images below the graph show the growth and coalescence of bubbles to recover the plastron. As the circuit is closed, small bubbles are formed which coalesce into larger bubbles until the plastron is recovered which blocks the conductive base and prevents further gas evolution.

Figure 5 shows the current transient as the plastron is recovered with images of the process shown below. A video of this process is available in the Supporting Information S1. We see that initially as the potential is applied and electrolysis begins, many small bubbles are generated on the conductive PDMS. As they grow, they start to coalesce into larger bubbles through an Oswald ripening type process until only two gas domains occupy each channel with a small area of base exposed between them. Due to reduced base area and blocking by the adjacent bubbles, the gas evolution slows down throughout the process with coalescence of the last two bubbles in each channel taking the longest to occur. When the channel is filled with gas, the base is fully blocked from the solution preventing the further passage of current. The average and standard deviation of time to dewet is 42 ± 11 s, average charge required is 367 ± 56 mC cm^–2^, and average energy 3.67 ± 0.56 J cm^–2^, based on three full plastron recoveries. Further example full dewetting data is available in the Supporting Information, Figures S1 and S2.

### Immediate Partial Plastron Recovery

**Figure 6:**
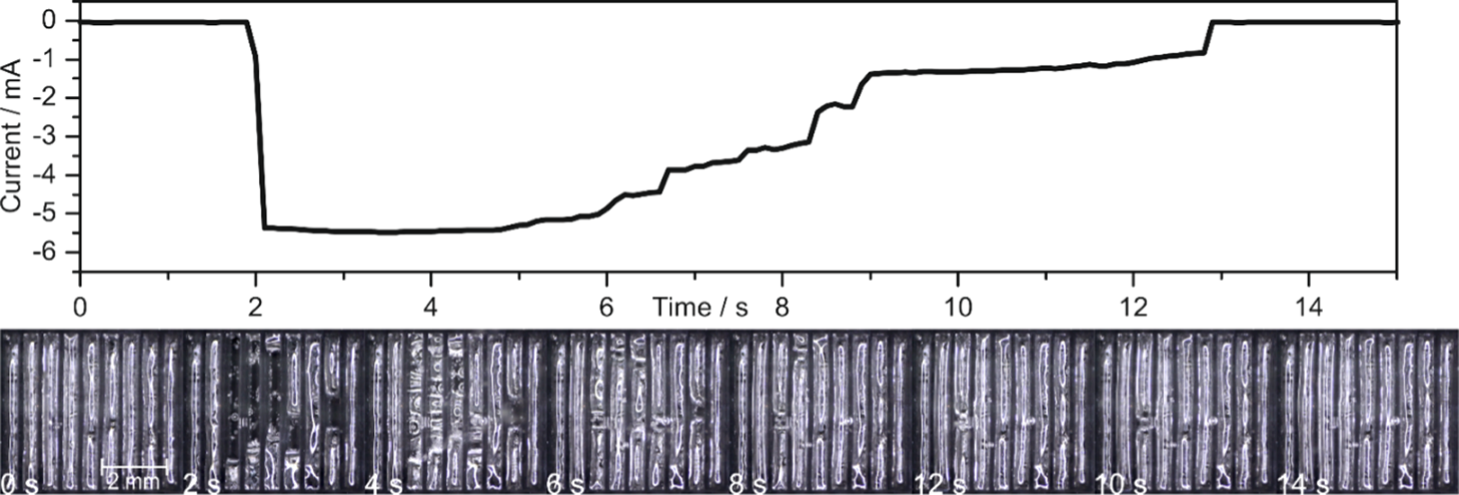
Immediate recovery when the plastron is displaced by a water jet and the surface held at a potential of −10 V. The graph shows the current transient, and images show the process below. Initially no current passes, but when the plastron is partially removed, the current increases, generating more gas which recovers the plastron.

The ability to restore a partially compromised plastron immediately after a pressure-induced wetting transition was also investigated. Figure 6 shows the current transient and images of the process, a video is available in the Supporting Information. Initially, the plastron is intact, the potential of the surface is held at −10 V, but no current is drawn as the plastron blocks the conductive base. At 2 s, the plastron is partially removed exposing the conductive base and immediately generating gas by electrolysis for 11 s before the plastron is fully recovered, returning the current to zero. Further example of immediate partial plastron recovery is available in the Supporting Information, Figure S3.

### Sensing of Wetting State

**Figure 7:**
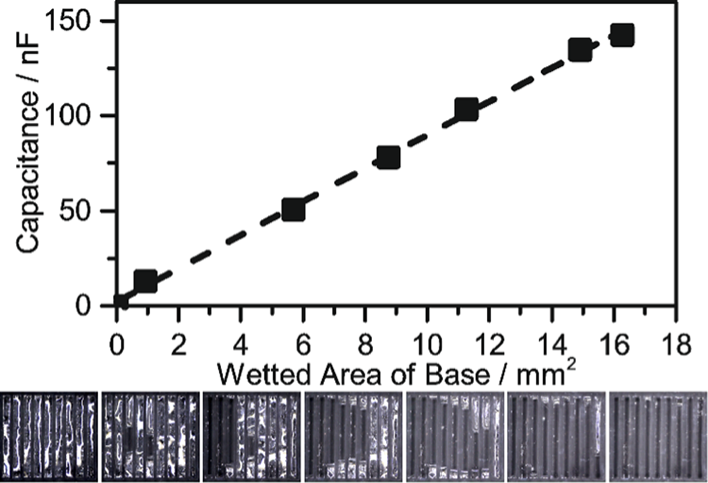
Sensing of the wetting state using capacitance measurement. By displacing the plastron in a stepwise manner, we observe a proportional relationship between wetted area and capacitance.

To demonstrate the remote measurement of the wetted state capacitance, measurements were used in a similar manner to our previous work.^18^ This method could be useful to quickly assess the integrity of the plastron without the need for a direct visual observation. To show the relationship between capacitance and wetted area, the surface was wetted in a stepwise fashion, removing small amounts of plastron over several steps and measuring the capacitance and the wetted area at the base of the channels from the macroscope image. Figure 7 shows a strong linear relationship between the two, where the gradient gives a capacitance density of 0.88 μF cm^–2^. The proportional scaling allows fast and accurate analysis of the wetting state without the need for direct visual access.

### Slip Length Measurement

The slip length of water over the surface was measured with a rheometry system using a 6 cm diameter cone, based on the method shown by Choi and Kim by converting the torque measurement to a slip length.^22^ A sample was fabricated with the same channel geometry as before (*p* = 600 μm, ϕ = 0.66) but in a concentric arrangement so that the channels were always arranged parallel to the flow. Support structures joining adjacent channels were introduced to channels longer than 13.4 mm to improve wall stability.

**Figure 8:**
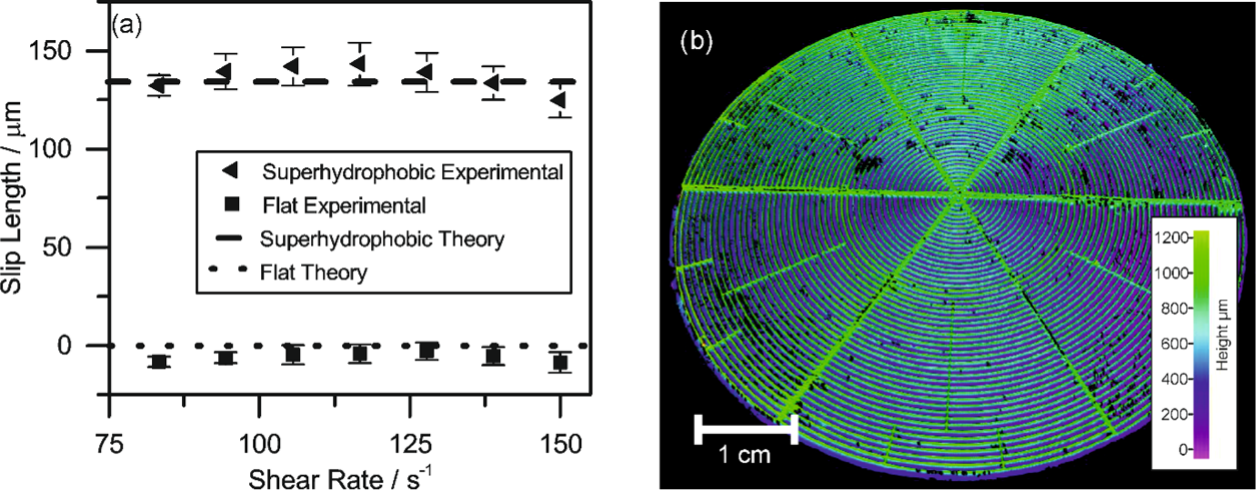
Measurement of slip length over the surface. (a) Good agreement between the experimental data, measured using a rheometry system and theory, using eq 2. Our surface produces a slip length of ∼135 μm compared to a flat surface, which holds the no-slip condition as expected. (b) Height image of the test sample used for slip measurements, 6 cm in diameter with concentric channels.

The slip length of water over a flat surface and over our superhydrophobic surface is shown against shear rate in Figure 8a, and a 3D confocal microscope image is shown for the sample used for the measurements in Figure 8b. The measured slip length shows some change with a shear rate as also seen by Choi and Kim^22^ and is ∼135 μm, which shows good agreement with eq 2. We see that the flat steel surface measured as a control holds the no-slip condition as expected. There was no difference in slip length between the “native” plastron and recovered plastron. This slip length is large enough to reduce drag in macrosized laminar flows with boundary layers of the order of millimeters. We would also expect a significant reduction to drag in turbulent flows, which also scales with pitch and gas fraction.^4^

### Flexibility and Damage Resistance

**Figure 9:**
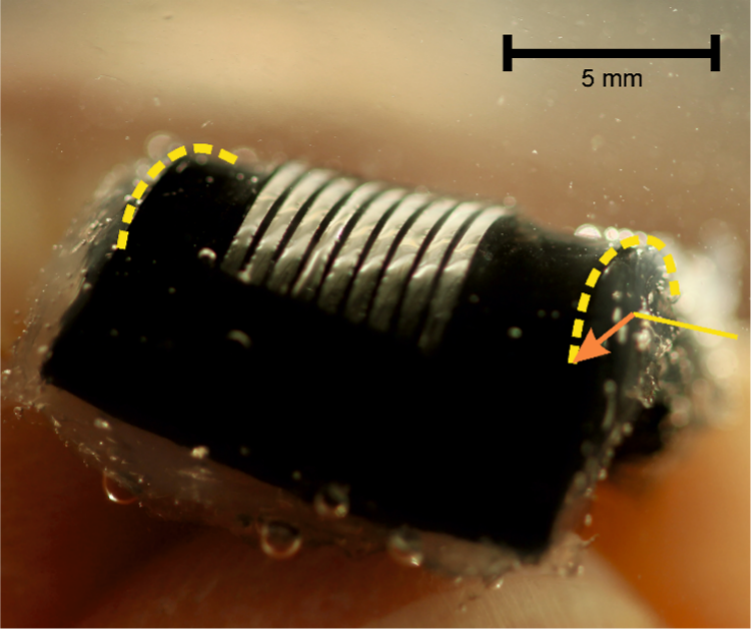
Demonstration of the flexibility of the surface. The photo shows a sample bent to a radius of curvature of ∼1 cm.

Due to the silicone-based construction, the surface can deform elastically without damage and return to its initial shape. Figure 9 shows a submersed sample bent to a radius of curvature of ∼1 cm. This property would allow the surface to conform to a wide range of shapes, e.g., the hull of a boat or a pipe without modification to the manufacture process. To simulate a collision with another body, we used an indentation test loading a 6 mm diameter steel sphere on the surface. We found that the loads of up to 50 N (load rate of 50 N s^–1^) could be withstood without damage or effect to the dewetting performance.

## Discussion and Conclusions

This work has shown that it is possible to recover the plastron from a truly fully wetted state and without the need for a superhydrophobic base, which has not previously been demonstrated. This means that there is no possibility of failed plastron recovery as in the case where a nanostructured superhydrophobic base is used.

Our surface has a significant slip length of 135 μm, large enough to reduce drag in laminar flows around macrosized objects. We would also expect a large reduction to drag in turbulent flows.

We have also demonstrated other important engineering aspects to this surface. The wetting state of the surface can be quickly measured through the analysis of the capacitance. The remote sensing of the plastron integrity would no doubt be important in real-world scenarios. We have also shown good damage resistance thanks to the silicone construction deforming elastically under load. This is an important consideration as minor collisions with debris in the ocean is a frequent occurrence. Hypothetically, the surface could be powered by cathodic protection systems already present in ships and pipes, which are used to prevent corrosion by holding the structures at cathodic potentials.

Further improvements to decrease the resistance of the base material could reduce the energy required to run such a system. The general approach presented in this work could be a viable route to bring sustained underwater drag reduction to ships and pipes.

## Experimental Methods

### Fabrication of Surface

The mold was manufactured by routing channels into lucite acrylic using a 200 μm diameter cutter.

A platinum curing silicone compound, Sylgard 184 by Dow, was used for the channel walls, in a 10:1 base/curing compound mix ratio. The Sylgard 184 was mixed by hand for 2 min and then degassed under vacuum to remove any bubbles. The mixture was applied to the mold using a flexible silicone spatula and spread to fully fill the channels and remove any excess from the top of the mold, so that the silicone in the channels was flush with the top of the mold. The mold was placed in an oven at 80 °C for 4 h to fully cure the silicone. After letting the mold to cool to room temperature, a lint-free polishing cloth was used to remove any small amount of silicone on the top of the mold between the channels filled with cured silicone.

The base comprised of the same Sylgard 184 in 10:1 mix ratio which was mixed by hand for 10 min with 20% by weight Vulcan XC-72R by Cabot Corp, a high electronic conductivity carbon black powder, until a homogenous mixture was obtained. The mixture was degassed under vacuum to remove any bubbles and then applied to the mold using a silicon spatula to coat silicone-filled channels fully at a thickness of around 2 mm. The plastic insulation from a stranded metal wire was stripped from the last 1 cm of the wire and the strands spread apart. The stripped end of the wire was placed on the uncured silicone and carbon black mixture, and further 2 mm of the mixture was added to fully encase the end of the wire. Samples were cured at a temperature of 80 °C for 4 h. After letting cool to room temperature, the sample was demoulded. To insulate the back and sides of the sample, Sylgard 184 in 10:1 mix ratio was carefully spread over these areas in a thin film, which was cured at a temperature of 80 °C for 4 h after which the sample was ready to use.

Contact angle measurements were made using a Krüss DSA100 contact angle apparatus.

### Dewetting and Capacitance Experiments

An Autolab PGSTAT302N with FRA32M module was used for all dewetting and capacitance measurements. The potential was measured against a platinum wire as a quasireference electrode. A large area platinum gauze electrode was used for the counter electrode. For capacitance measurements, electrochemical impedance spectroscopy was used by scanning 10 frequencies between 1000 and 10000 Hz with a potential modulation of ±5 mV about a potential of −0.4 V vs Pt wire reference. A Randle’s circuit was fitted to the data from which the capacitance was obtained.

### Rheometry

The rheometer used was an AR2000 by TA Instruments. Before the measurements were taken, a full calibration was undertaken and confirmed with the testing of a reference oil. The same precautions were taken to reduce the errors discussed as in Choi and Kim.^23^ A cone of 6 cm diameter, 2° cone angle, and 53 μm truncation was used. The temperature-controlled plate was held at 20 °C.

## Supporting Information Available

Graph showing repeat data for full plastron recoveries, and a graph showing multiple partial plastron recoveries.(PDF)
Video showing full dewetting and immediate partial plastron recovery (MP4)
