# Supplementary material for: Complete Electrolytic Plastron Recovery in a Low Drag Superhydrophobic Surface
Source: ACS Omega. 2021 Jan 28;6(5):3483–9. doi: 10.1021/acsomega.0c03466 (PMC7906494; doi:10.1021/acsomega.0c03466)
Supplement: Supplementary file 1 — ao0c03466_si_001.pdf [file ao0c03466_si_001.pdf]

Supporting Information for: Complete Electrolytic Plastron Recovery in a Low Drag Superhydrophobic Surface

Ben P. Lloyd†, Philip N. Bartlett‡, Robert J. K. Wood†*

†National Centre for Advanced Tribology at Southampton (nCATS), University of Southampton, SO17 1BJ, UK

‡Chemistry, University of Southampton, SO17 1BJ, UK

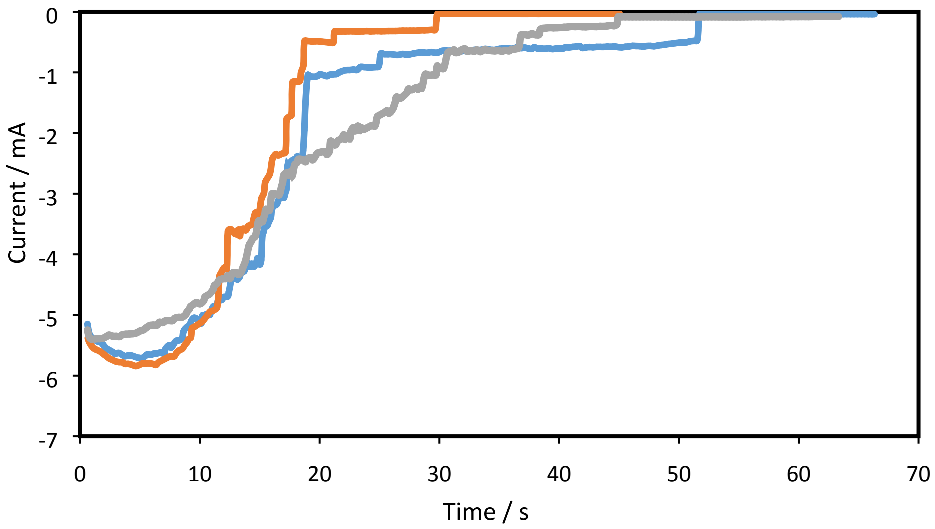

Figure S1: Current transients for repeated dewetting of the surface from a fully wetted state, typical of all surfaces tested. A potential of -10 V was applied to the surface.

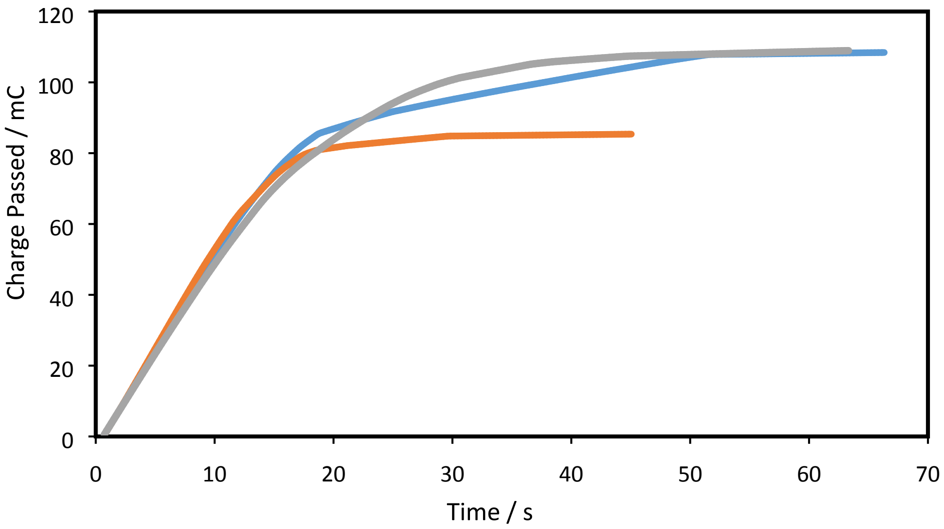

Figure S2: The data from Figure S1 integrated with respect to time to give the charge passed

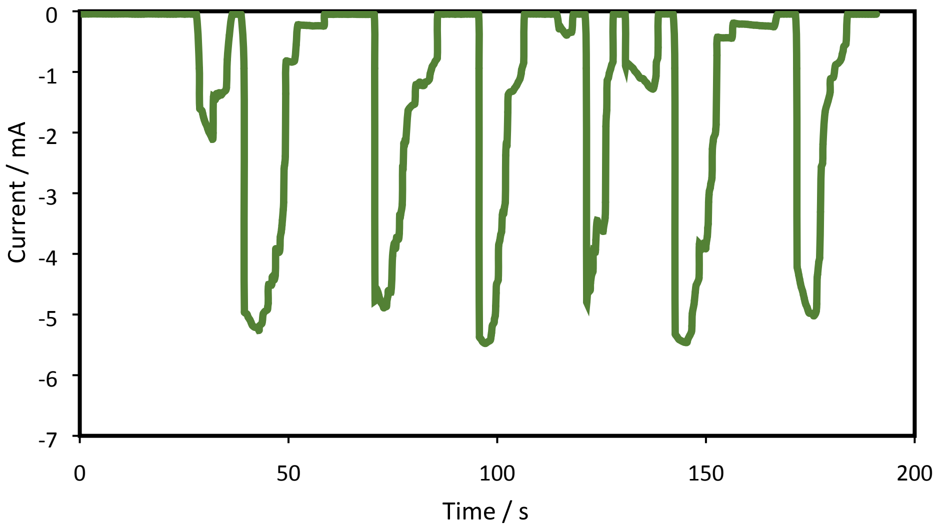

Figure S3: Example of repeated partial dewetting events. The surface is held at -10 V and initially has the full plastron intact, therefore no current is passed. The plastron is partially displaced by using a pipette to create a water jet. The current returns to zero as the plastron is recovered. The process was repeated 9 times to show the repeatability of the partial dewetting behaviour.
